# DDR2 overexpression in urothelial carcinoma indicates an unfavorable prognosis: a large cohort study

**DOI:** 10.18632/oncotarget.12912

**Published:** 2016-10-26

**Authors:** Meng-Chen Tsai, Wei-Ming Li, Chun-Nung Huang, Hung-Lung Ke, Ching-Chia Li, Hsin-Chih Yeh, Ti-Chun Chan, Peir-In Liang, Bi-Wen Yeh, Wen-Jeng Wu, Sher-Wei Lim, Chien-Feng Li

**Affiliations:** ^1^ Department of Pathology, Chi-Mei Medical Center, Tainan, Taiwan; ^2^ Graduate Institute of Medicine, College of Medicine, Kaohsiung Medical University, Kaohsiung, Taiwan; ^3^ Department of Urology, Kaohsiung Medical University Hospital, Kaohsiung, Taiwan; ^4^ Department of Urology, School of Medicine, College of Medicine, Kaohsiung Medical University, Kaohsiung, Taiwan; ^5^ Department of Urology, Ministry of Health and Welfare Pingtung Hospital, Pingtung, Taiwan; ^6^ Department of Urology, Kaohsiung Municipal Ta-Tung Hospital, Kaohsiung, Taiwan; ^7^ Department of Pathology, Kaohsiung Medical University Hospital, Kaohsiung, Taiwan; ^8^ Center for Infectious Disease and Cancer Research, Kaohsiung Medical University, Kaohsiung, Taiwan; ^9^ Center for Stem Cell Research, Kaohsiung Medical University, Kaohsiung, Taiwan; ^10^ Institute of Medical Science and Technology, National Sun Yat-sen University, Kaohsiung, Taiwan; ^11^ Institute of Biomedical Sciences, National Sun Yat-sen University, Kaohsiung, Taiwan; ^12^ Department of Neurosurgery, Chi-Mei Medical Center, Chiali, Tainan, Taiwan; ^13^ Department of Nursing, Min-Hwei College of Health Care Management, Tainan, Taiwan; ^14^ Department of Biotechnology, Southern Taiwan University of Science and Technology, Tainan, Taiwan; ^15^ National Cancer Research Institute, National Health Research Institutes, Tainan, Taiwan; ^16^ Institute of Clinical Medicine, Kaohsiung Medical University, Kaohsiung, Taiwan; ^17^ Department of Internal Medicine and Cancer Center, Kaohsiung Medical University Hospital, Kaohsiung Medical University, Kaohsiung, Taiwan

**Keywords:** urothelial carcinoma, transcriptome, DDR2, prognosis

## Abstract

The migration ability of urothelial carcinoma corresponding to dismal prognosis had not been fully investigated. The interaction of extracellular collagen with a unique transmembrane receptor tyrosine kinase, Discoidin domain receptor 2 (DDR2), was selected by data mining. We arranged real-time reverse transcription polymerase chain reaction assays to evaluate the transcript levels in 26 urinary tract urothelial carcinoma and 26 urinary bladder urothelial carcinoma specimens, showing significantly increase corresponding to advanced primary stage (*p* = 0.003 and *p* < 0.001, respectively). An immunohistochemistry analysis and H-score calculation were performed to determine DDR2 expression in 340 urinary tract urothelial carcinoma and 295 urinary bladder urothelial carcinoma. Assessments of the correlation to clinicopathologic features, disease-specific survival, and metastasis-free survival were conducted. The transcript levels in advanced stage were higher than those in early stage and were correlated with poor prognosis. The higher expression was positively correlated to higher pT status (*p* < 0.001), higher histological grade (urinary tract, *p* = 0.041; urinary bladder, *p* < 0.001), greater vascular invasion (*p* < 0.001), and higher mitotic rate (urinary tract, *p* = 0.039; urinary bladder, *p* < 0.001). Higher expression also indicates significantly worse disease-specific survival and metastasis-free survival. In vitro study revealed knockdown of DDR2 resulted in a depletion of cellular viability, migratory, and invasive ability, supporting the oncogenic function of DDR2.

## INTRODUCTION

Urothelial carcinoma (UC), a most common cancer from the urinary bladder and upper tract, features complex gene expression and molecular interactions [1]. Based on the database of the Taiwan Cancer Registry in 2012, the age-standardized incidence rate of bladder malignancy was 8.70 and 3.34 per 100000 persons in males and in females, respectively, and the age-standardized mortality rate for bladder cancer was 3.08 and 1.34 per 100000 persons in males and in females, respectively [2]. Among the initial diagnosis, approximately one third of patients have invasive disease or metastatic event [1]. Though advances in chemotherapy for patients with advanced UC have been achieved, most of these patients will develop resistance to treatment [4]. The combination of cisplatin and gemcitabine is the first-line treatment for metastatic UC, but the response rate is 50% actually, with a median progression free survival of 7 to 8 months [5]. Hence, there is a need not only to investigate the cellular signaling pathways involved in UC, but also to discover prognostic markers and therapeutic targets.

In this study, we investigate the relationship between UC and a special receptor tyrosine kinase (RTK) activated by collagen in the extracellular matrix, named Discoidin domain receptor 2 (DDR2) [6]. The receptor gains its unique place by functioning as a sensor for collagen and by participating in migration, proliferation, and extracellular matrix remodeling. The expression of DDR2 had been previously observed in the development of tissue, homeostasis, response to injury, and tumorigenesis [7, 8].

To our knowledgement, the role of DDR2 in UC have never been investigated before. Via data mining, we identified upregulation of DDR2 among transmembrane receptor protein tyrosine kinase in advanced UC. We further investigated RNA transcription level using real-time RT-PCR, and protein expression intensity evaluated by immunohistochemistry study, and the correlation to clinicopathologic parameters and survival.

## RESULTS

### *DDR2* was recognized as a significantly overexpressed transcript in invasiveness and metastasis in UBUC

**Figure 1:**
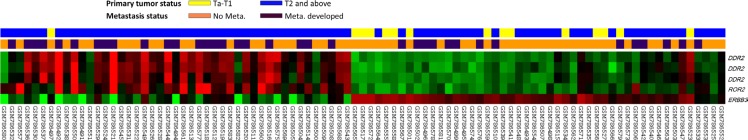
Gene expression profile analysis in urinary bladder urothelial carcinoma from a published transcriptomic dataset (GSE31684) Clustering analysis of genes regarding transmembrane receptor protein tyrosine kinase activity showed DDR2 as the most up-regulated gene with both higher primary tumor status (pT) and distal metastasis. Samples from the high pT (T2 and above, blue lines), low pT (Ta and T1, yellow lines), metastasis (purple lines), and absence of metastasis (orange lines) are shown on top of the heatmap, and the upregulation and downregulation of mRNA transcriptional level are displayed as a spectrum of brightness of red and green, respectively. The unaltered ones are coded black.

Reanalysis of the transcriptomic profile from GSE31684 with special attention to those associated with transmembrane receptor protein tyrosine kinase activity (GO:0004714), three transcripts were identified to have significant differential expression (Figure 1). These include upregulation of *DDR2* and *ROR2* and down-regulation of *ERBB3*. Among them, *DDR2* is the most significantly upregulated that showed log2 ratios of 0.9193-fold and 0.8109-fold upregulation related to the increment of primary tumor (pT) status and presence of metastasis (both *P* < 0.0001, Table 1). More importantly, the expression level of *DDR2* transcripts, comparing high-expression (*n* = 37) to low-expression (*n* = 56) clusters, significantly predicted disease-specific survival (Figure 2, *P* = 0.0335). Given that DDR2 has not been systemically studied in UCs, prompting us to further characterize its clinical significance in UC.

**Table 1:** Summary of differentially expressed genes associated with transmembrane receptor protein tyrosine kinase activity (GO:0004714) and showed positive associations to cancer invasiveness and metastasis in the transcriptome of urothelial carcinoma of urinary bladder (GSE31684)

Probe	Comparing T2–4 to Ta-T1		Comparing Meta.to Non-Meta.^#^		Gene Symbol	Biological Process	Molecular Function
	log ratio	*p*-value	log ratio	*p*-value			
205168_at	0.6233	0.0077	0.5747	0.0018	***DDR2***	cell adhesion, positive regulation of cell proliferation, protein amino acid phosphorylation, signal transduction, transmembrane receptor protein tyrosine kinase signaling pathway	ATP binding, kinase activity, nucleotide binding, protein kinase activity, protein-tyrosine kinase activity, receptor activity, transferase activity, transmembrane receptor protein tyrosine kinase activity
205578_at	0.6393	<0.0001	0.3795	0.0010	***ROR2***	JNK cascade, Wnt receptor signaling pathway; calcium modulating pathway, cartilage condensation, cell differentiation, embryonic genitalia morphogenesis, multicellular organismal development, protein amino acid phosphorylation, signal transduction, skeletal development, somitogenesis	ATP binding, kinase activity, nucleotide binding, protein binding, protein kinase activity, protein-tyrosine kinase activity, receptor activity, transferase activity, transmembrane receptor protein tyrosine kinase activity
225442_at	0.9193	0.0001	0.8109	<0.0001	***DDR2***	cell adhesion, positive regulation of cell proliferation, protein amino acid phosphorylation, signal transduction, transmembrane receptor protein tyrosine kinase signaling pathway	ATP binding, nucleotide binding, protein-tyrosine kinase activity, receptor activity, transferase activity, transmembrane receptor protein tyrosine kinase activity
227561_at	0.796	0.0011	0.7064	0.0002	***DDR2***	cell adhesion, positive regulation of cell proliferation, protein amino acid phosphorylation, signal transduction, transmembrane receptor protein tyrosine kinase signaling pathway	ATP binding, nucleotide binding, protein-tyrosine kinase activity, receptor activity, transferase activity, transmembrane receptor protein tyrosine kinase activity
226213_at	−2.2339	<0.0001	−0.8044	0.0036	***ERBB3***	heart development, peripheral nervous system development, protein amino acid phosphorylation, signal transduction, transmembrane receptor protein tyrosine kinase signaling pathway	ATP binding, epidermal growth factor receptor activity, kinase activity, nucleotide binding, protein binding, protein heterodimerization activity, protein kinase activity, protein-tyrosine kinase activity, receptor activity, transferase activity, transmembrane receptor protein tyrosine kinase activity

# Meta., distal metastasis developed during follow-up; Non-Meta.: no metastatic event developed.

**Figure 2:**
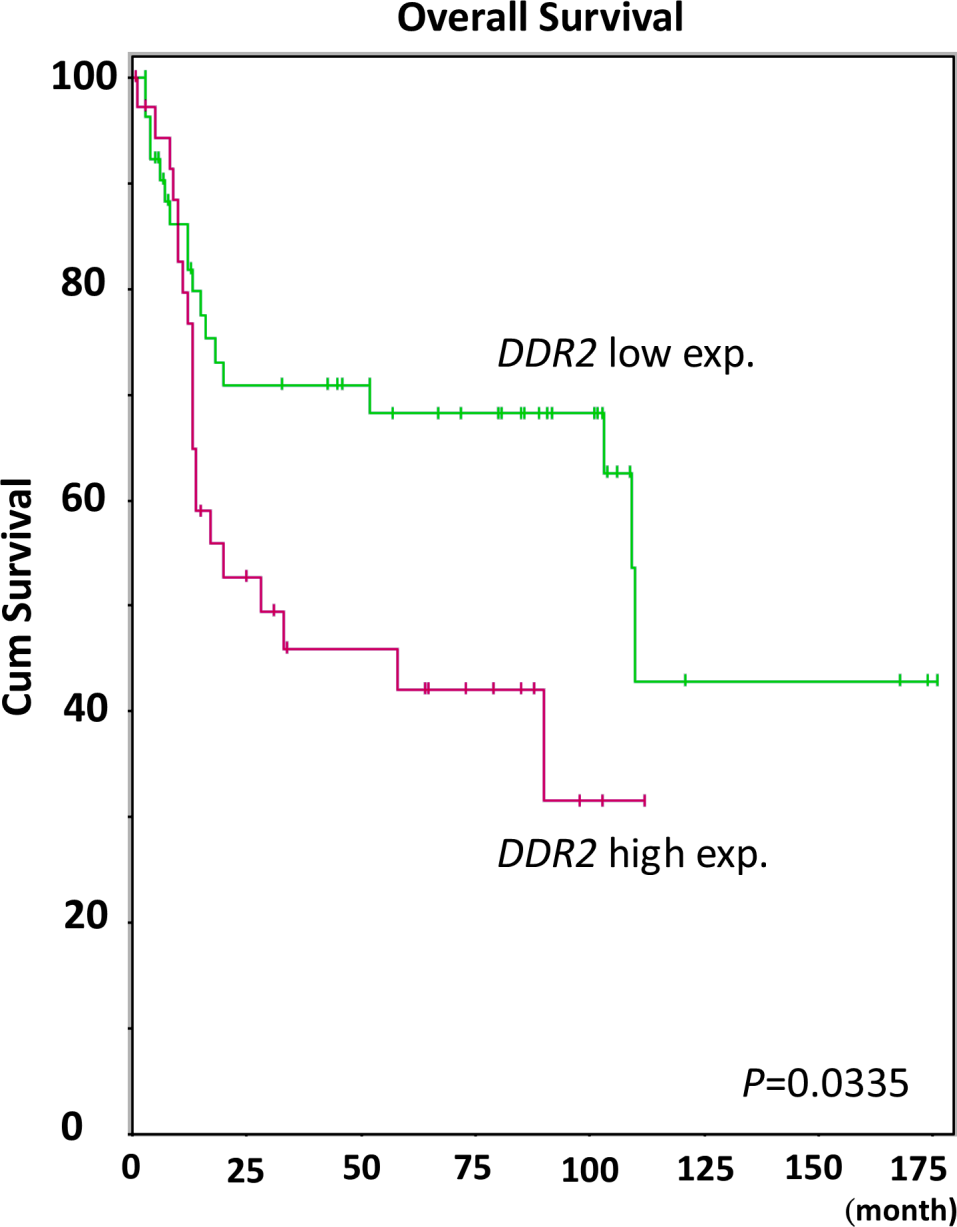
Kaplan-Meier plot generated from GSE31684 reveals the prognostic significance of DDR2 expression level for the overall survival of urothelial carcinoma by comparing 37 cases with high DDR2 expression and 56 with low expression (*P* = 0.0335)

### *DDR2* mRNA expression is positively correlated with advanced pT status in UTUC and UBUC

**Figure 3:**
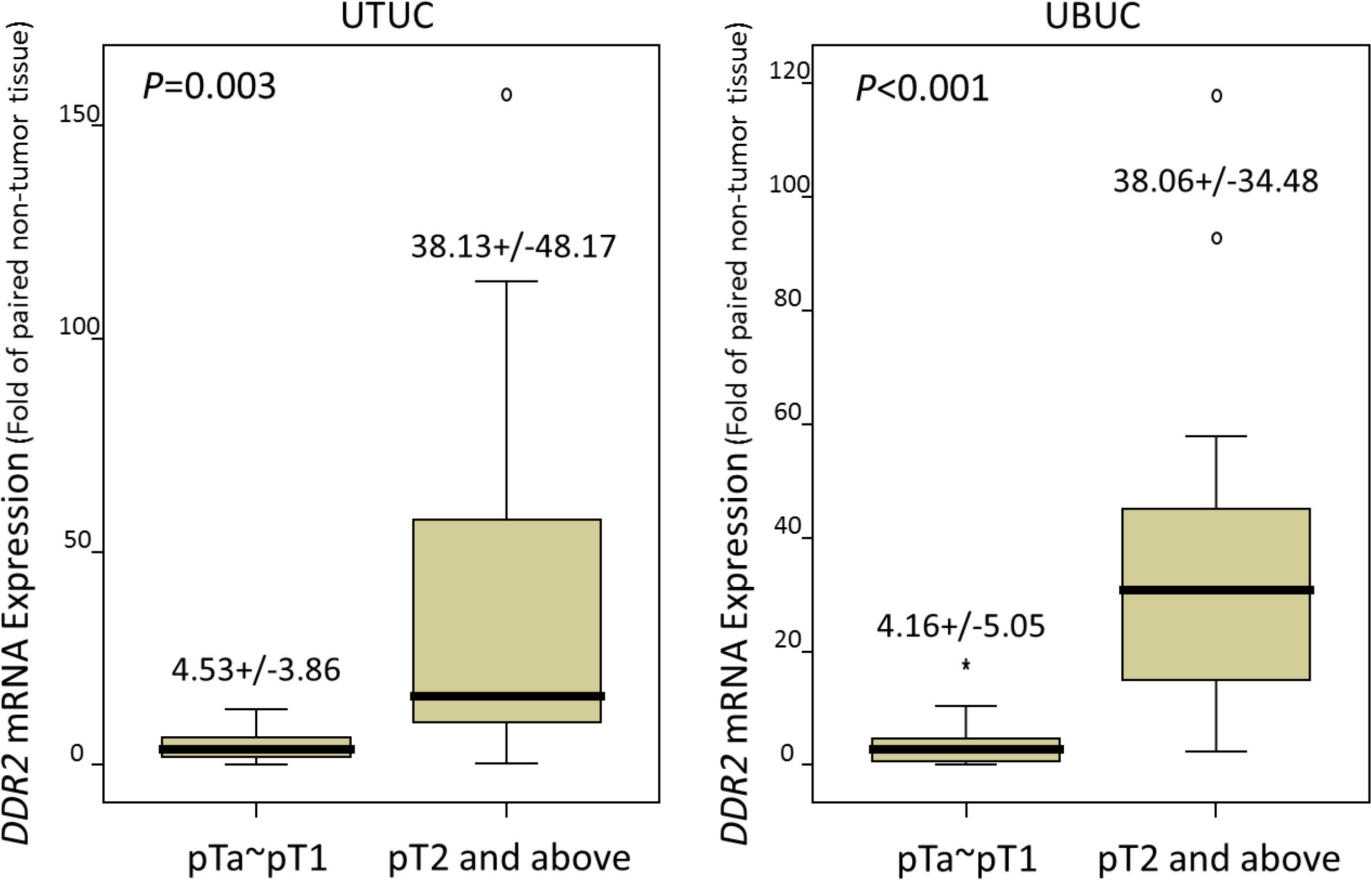
Quantitative real-time RT-PCR analysis discovers that *DDR2* mRNA is significantly overexpressed in urinary tract urothelial carcinomas (UTUCs, *left* panel) and urinary bladder urothelial carcinomas (UBUCs, *right* panel) with higher primary tumor statuses

*DDR2* mRNA expression was significantly elevated with advanced pT status in the 26 UTUCs (*P* = 0.003) and 26 UBUCs (*P* < 0.001), confirming that DDR2 participated in tumor progression (Figure 3)

### Clinicopathologic findings of UTUC

Table 2 lists the clinical and pathological data of the UTUC patients. The age of diagnosis was between 34 and 87 years, and the median age was 68 years. One hundred and thirty-eight patients (40.6%) were diagnosed under 65 years of age. Sixty-two patients (18.2%) had multifocal tumors, and 49 (14.4%) had both renal pelvis and ureter tumors. Approximately half of the cases (46.8%) presented as advanced pT stage (pT2–T4). Twenty-eight patients (8.2 %) had lymph node metastasis. Most of the tumors (*n* = 284, 83.5 %) showed a high histological grade. Frequent mitosis defined by over 10 per 10 high power fields was observed in 49.1% of the cases. Vascular invasion and perineurial invasion presented in 31.2% and 5.6% of cases, respectively.

**Table 2:** Correlations between DDR2 expression and other important clinicopathological parameters in urothelial carcinomas

Parameter	Category	Upper Carcinoma	Urinary	Tract	Urothelial	Urinary	Bladder	Urothelial	Carcinoma
		Case No.	DDR2 Expression		*p*-value	Case No.	DDR2 Expression		*p*-value
			Low	High			Low	High	
Gender	Male	158	81	77	0.664	216	110	106	0.667
	Female	182	89	93		79	38	41	
Age (years)	< 65	138	67	71	0.659	121	63	58	0.587
	≥ 65	202	103	99		174	85	89	
Tumor location	Renal pelvis	141	63	78	0.250	−	−	−	−
	Ureter	150	80	70		−	−	−	−
	Renal pelvis & ureter	49	27	22		−	−	−	−
Multifocality	Single	278	137	141	0.574	−	−	−	−
	Multifocal	62	33	29		−	−	−	−
Primary tumor (T)	Ta	89	60	29	< 0.001*	84	63	21	< 0.001*
	T1	92	58	34		88	43	45	
	T2-T4	159	52	107		123	42	81	
Nodal metastasis	Negative (N0)	312	161	151	0.049*	266	136	130	0.319
	Positive (N1-N2)	28	9	19		29	12	17	
Histological grade	Low grade	56	35	21	0.041*	56	44	12	< 0.001*
	High grade	284	135	149		239	104	135	
Pattern of Invasion	Nodular	200	124	76	< 0.001*	137	87	50	< 0.001*
	Trabecular	58	21	37		82	37	45	
	Infiltrative	82	25	57		76	24	52	
Vascular invasion	Absent	234	142	92	< 0.001*	246	135	111	< 0.001*
	Present	106	28	78		49	13	36	
Perineural invasion	Absent	321	166	155	0.009*	275	142	133	0.062
	Present	19	4	15		20	6	14	
Mitotic rate (per 10 high power fields)	< 10	173	96	77	0.039*	139	92	47	< 0.001*
	>= 10	167	74	93		156	56	100	

* Statistically significant.

### Clinicopathologic features of UBUCs

In the UBUC group, the majority of patients were male (73.2%). One hundred and twenty-one patients (41.0%) were diagnosed under 65 years of age. About half of the cases (41.6%) presented at an advanced pT stage (pT2–T4). Nodal metastasis was detected in 23.6% (*n* = 29) of the cases. The vast majority of the tumors (81%) showed a high histological grade. Cases with frequent mitosis (52.9%) slightly outnumbered those that did not have frequent mitosis. Vascular invasion and perineurial invasion presented in 16.7% and 6.8% of cases, respectively.

### Correlations between immunoreactivity of DDR2 and clinicopathologic parameters in UTUCs and UBUCs

**Figure 4:**
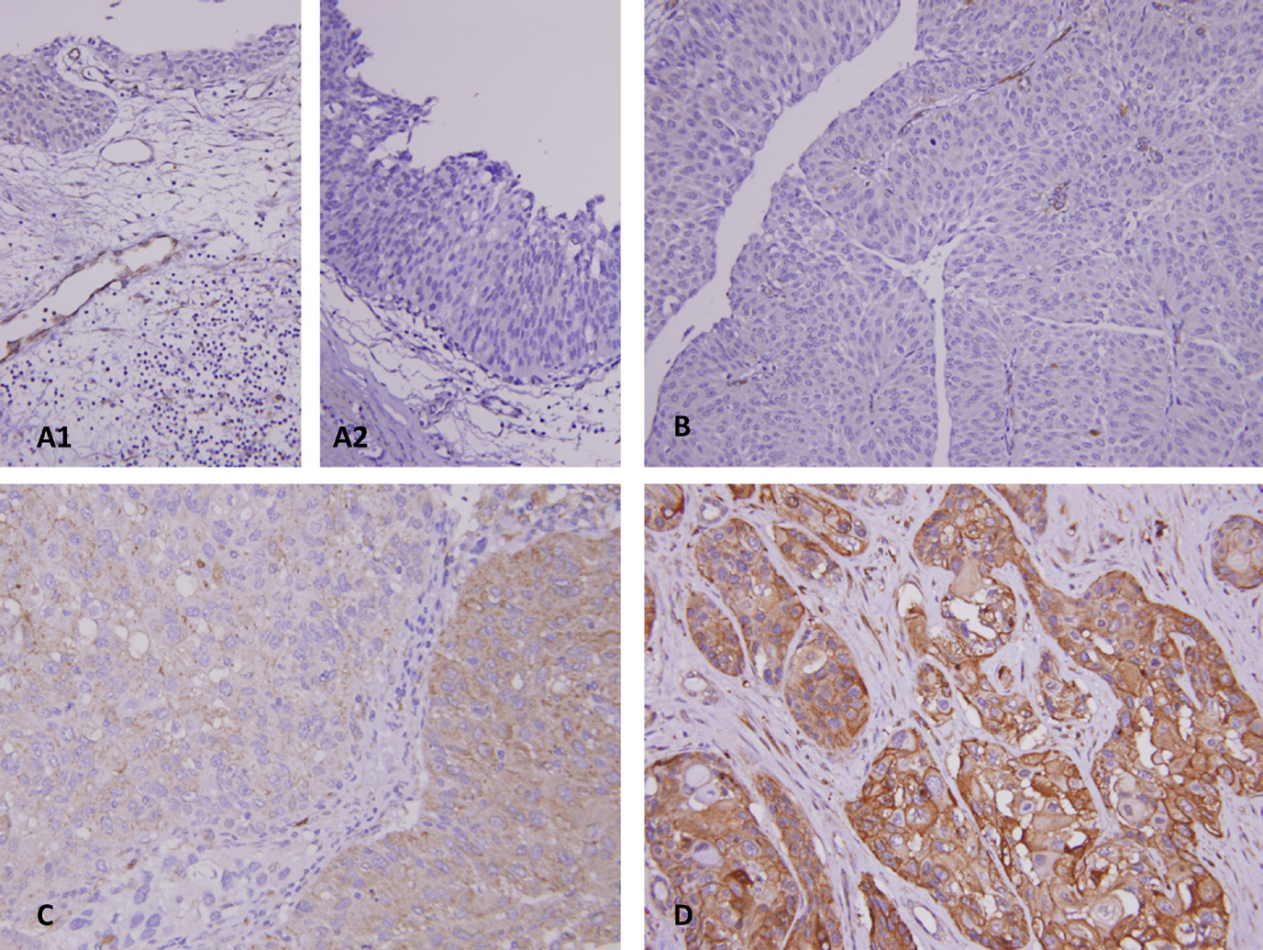
Representative lesions for DDR2 immunostaining reveals DDR2 is undetected in normal urothelium (**A1**), and urothelial dysplasia (**A2**), and non-invasive papillary urothelial carcinoma (**B**). There is an escalated increase of immunoacitvity from superficial invasive (**C**) to muscle invasive (**D**) urothelial carcinoma.

As described in Table 2, after dichotomizing DDR2 immunoactivity into low and high levels, increased expression in UC of the upper urinary tract and urinary bladder was significantly linked to several clinicopathologic parameters, including an advanced pT status (Figure 4, UTUC and UBUC, *P* < 0.001), high grade histological patterns (UTUC, *P* = 0.041; UBUC, *P* < 0.001), vascular invasion (UTUC and UBUC,*P* < 0.001), and higher mitotic rate (UTUC, *P* = 0.039; UBUC, *P* < 0.001). Increased DDR2 expression was significantly associated with nodal metastasis and perineurial invasion only in the UTUC group (*P* = 0.049 and 0.009, respectively). More than half of the tumor shows infiltrative and trabecular invasion pattern in both UTUC and UBUC groups, significantly corresponding to high DDR2 expression (UTUC and UBUC, *P* < 0.001).

### Survival analysis in patients with UTUCs and UBUCs

The association between clinical outcomes and clinicopathologic parameters is assessed by the univariate and multivariate analyses in UTUC and UBUC patients, as illustrated in Tables 3 and 4, respectively. In the multivariate analysis of the UTUC group, inferior DSS was significantly associated with multifocality (*P* = 0.010), primary tumor status (*P* = 0.033), nodal metastasis (*P* < 0.001), high histological grade (*P* = 0.044), and perineural invasion (*P* < 0.001). Likewise, multifocality (*P* = 0.010), nodal metastasis (*P* = 0.001), high histological grade (*P* = 0.023), vascular invasion (*P* = 0.008) and perineural invasion (*P* = 0.008) were independent prognostic factors for a worse MeFS outcome. Tumor location was only significantly associated with worse DSS in univariate analysis (*P* = 0.0079). An advanced pT status was significantly associated with poor DSS in both univariate and multivariate analyses, but MeFS was significantly associated only in univariate (*P* < 0.0001) analysis. Vascular invasion correlated with poorer DSS only in univariate analysis (*P* < 0.0001) but was significantly associated with worse MeFS in both univariate and multivariate analyses.

**Table 3:** Univariate log—rank and multivariate analyses for disease-specific and metastasis-free survivals in upper urinary tract urothelial carcinoma

Parameter	Category	Case No.	Disease-specific Survival				Metastasis-free Survival					
			Univariate analysis		Multivariate analysis		Univariate analysis			Multivariate analysis		
			No. of event	*p*-value	R.R.	95% C.I.	*p*-value	No. of event	*p*-value	R.R.	95% C.I.	*p*-value
Gender	Male	158	28	0.8286	−	−	−	32	0.7904	−	−	−
	Female	182	33		−	−	−	38		−	−	−
Age (years)	< 65	138	26	0.9943	−	−	−	30	0.8470	−	−	−
	≥ 65	202	35		−	−	−	40		−	−	−
Tumor side	Right	177	34	0.7366	−	−	−	38	0.3074	−	−	−
	Left	154	26		−	−	−	32		−	−	−
	Bilateral	9	1		−	−	−	0		−	−	−
Tumor location	Renal pelvis	141	24	0.0079^*^	1	−	0.822	31	0.0659	−	−	−
	Ureter	150	22		0.712	0.200–2.538		25		−	−	−
	Renal pelvis & ureter	49	15		0.848	0.217–3.308		14		−	−	−
Multifocality	Single	273	48	0.0026^*^	1	−	0.010`^*^	52	0.0127^*^	1	−	0.010^*^
	Multifocal	62	18		2.774	1.274–6.039		18		2.569	**1.480–4.460**	
Primary tumor (T)	Ta	89	2	< 0.0001^*^	1	−	0.033^*^	4	< 0.0001^*^	1	−	0.260
	T1	92	9		3.919	0.832–18.450		15		3.314	1.072–10.248	
	T2-T4	159	50		5.163	1.137–23.439		51		2.729	0.969–8.580	
Nodal metastasis	Negative (N0)	312	42	< 0.0001^*^	1	−	< 0.001^*^	55	< 0.0001^*^	1	−	0.001^*^
	Positive (N1–N2)	28	19		5.178	2.837–9.450		15		2.877	**1.564–5.292**	
Histological grade	Low grade	56	4	0.0215^*^	1	−	0.044^*^	3	0.0027^*^	1	−	**0.023**
	High grade	284	57		2.964	1.030–8.534		67		3.926	**1.208–12.758**	
Vascular invasion	Absent	234	24	< 0.0001^*^	1	−	**0.176**	26	< 0.0001^*^	1	−	0.008^*^
	Present	106	37		1.504	0.833–2.717		44		2.237	**1.230–4.069**	
Perineural invasion	Absent	321	50	< 0.0001^*^	1	−	< 0.001^*^	61	< 0.0001^*^	1	−	0.008^*^
	Present	19	11		3.773	1.804–7.894		9		2.749	**1.295–5.835**	
Mitotic rate (per 10 high power fields)	< 10	173	27	0.167	−	−		30	**0.0823**	−	−	
	>= 10	167	34		−	−		40		−	−	
DDR2 expression	Low	170	13	< 0.0001^*^	1	−	**0.003**	15	< 0.0001^*^	1	−	< 0.001^*^
	High	170	48		2.637	1.404–4.949		55		2.797	**1.572–4.975**	

* Statistically significant

**Table 4:** Univariate log-rank and multivariate analyses for disease-specific and metastasis-free survivals in urinary bladder urothelial carcinoma

Parameter	Category	Case No.	Disease-specific Survival				Metastasis-free Survival					
			Univariate analysis		Multivariate analysis		Univariate analysis			Multivariate analysis		
			No. of event	*p*-value	R.R.	95% C.I.	*p*-value	No. of event	*p*-value	R.R.	95% C.I.	*p*-value
Gender	Male					216	41	0.4446	−	−	−	60	0.2720	−	−	−
	Female		79	11		−	−	−	16		−	−	−
Age (years)	< 65					121	17	0.1136	−	−	−	31	0.6875	−	−	−
	≥ 65		174	35		−	−	−	45		−	−	−
Primary tumor (T)	Ta					84	1	< 0.0001*	**1**	−	< 0.001*	4	< 0.0001*	**1**	**−**	0.001*
	T1		88	9		**6.361**	**0.702–57.605**		23		**5.107**	**1.512–17.245**	
	T2-T4		123	42		**27.516**	**2.800–218.273**		49		**7.401**	**2.173–25.205**	
Nodal metastasis	Negative (N0)					266	41	0.0002*	1	−	0.182	61	< 0.0001*	**1**	**−**	0.028*
	Positive (N1-N2)		29	11		1.211	0.799–3.254		15		**1.857**	**1.077–3.618**	
Histological grade	Low grade					56	2	0.0013*	1	−	0.788	5	0.0007*	1	−	0.888
	High grade		239	50		2.066	0.172–3.805		71		1.468	0.380–3.054	
Vascular invasion	Absent					246	37	0.0024*	1	−	0.157	54	0.0001*	1	−	0.675
	Present		49	15		1.608	0.307–1.209		22		1.055	0.488–1.590	
Perineural invasion	Absent					275	44	0.0001*	**1**	**−**	0.088	66	0.0007*	1	−	0.167
	Present		20	8		**2.495**	**0.897–4.824**		10		1.791	0.803–3.548	
Mitotic rate (per 10 high power fields)	< 10					139	12	< 0.0001*	**1**	**−**	0.037*	23	< 0.0001*	**1**	**−**	0.034*
	>= 10		156	40		**2.55**	**1.043–3.956**		53		**1.868**	**1.042–2.907**	
DDR2 expression	Low					147	10	< 0.0001*	**1**	**−**	0.001*	23	0.0001*	**1**	**−**	0.008*
	High	148	42		**3.488**	**1.712–7.108**		53		**2.006**	**1.203–3.345**	

* Statistically significant.

In multivariate analysis for patients with UBUC, advanced pT status and higher mitotic rate were independently associated with inferior DDS and MeFS (*P* < 0.05). Nodal metastasis was proven to be significantly and independently associated with worse MeFS (*P* = 0.028). Nodal metastasis, higher histological grade, vascular invasion, and perineural invasion correlated with adverse DSS only in univariate analysis. Similarly, higher histological grade, vascular invasion, and perineural invasion showed a significant association with MeFS only in univariate analysis.

### Prognostic significance of DDR2 expression in UC

**Figure 5:**
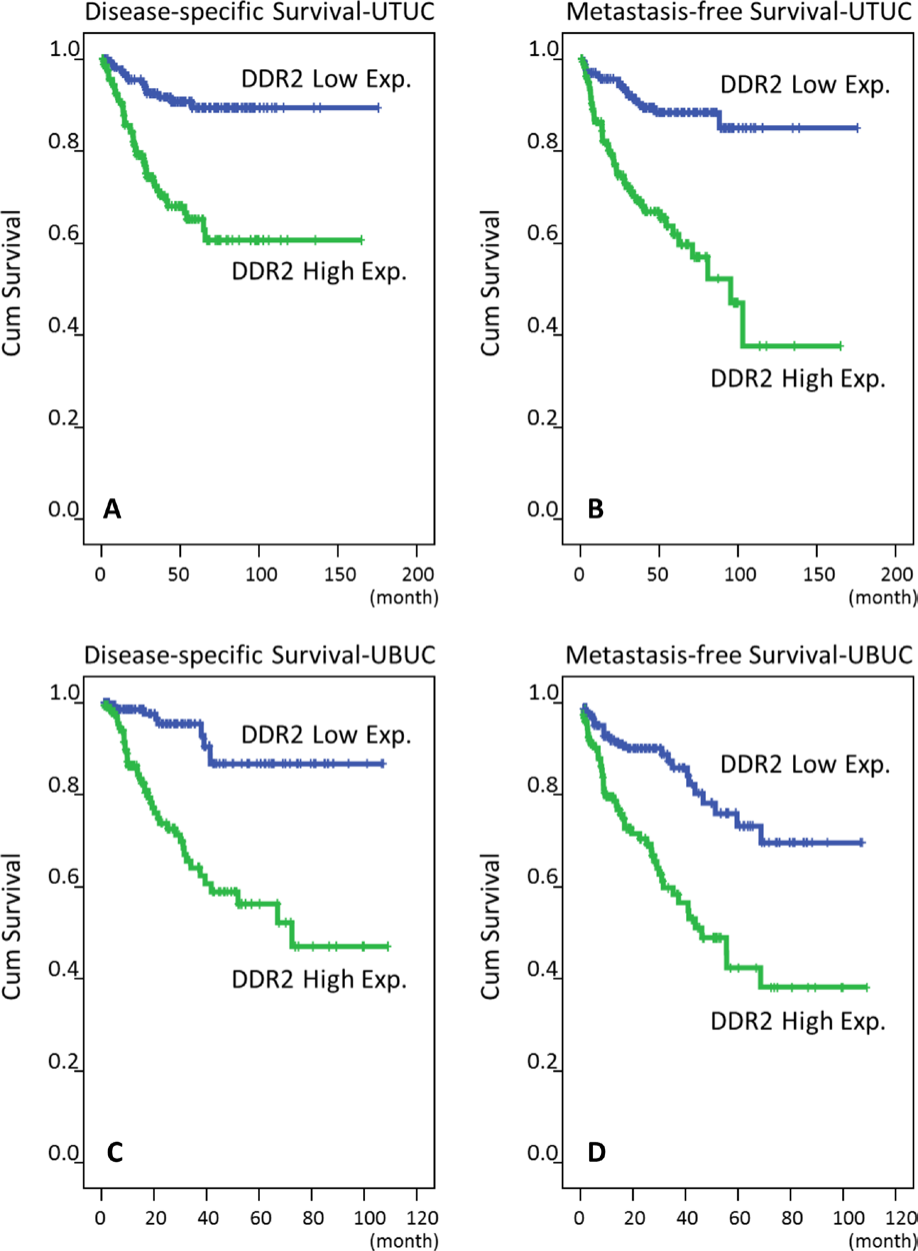
Survival analysis is depicted by Kaplan-Meier curves Proven by log-rank tests, high expression of DDR2 is predictive for worse disease-specific survival in both UTUC and UBUC (**A, C**) and for poor metastasis-free survival in both UTUC and UBUC (**B, D**), respectively.

As shown in Tables 3 and 4, in univariate and multivariate analyses, both the UTUC and UBUC group with high DDR2 expression had significantly dismal DSS and MeFS (*P* < 0.01 for all). Similar results were also noted in the Kaplan-Meier plots (Figure 5).

### DDR2 promotes proliferative, migratory, and invasive ability of UC cell lines

**Figure 6:**
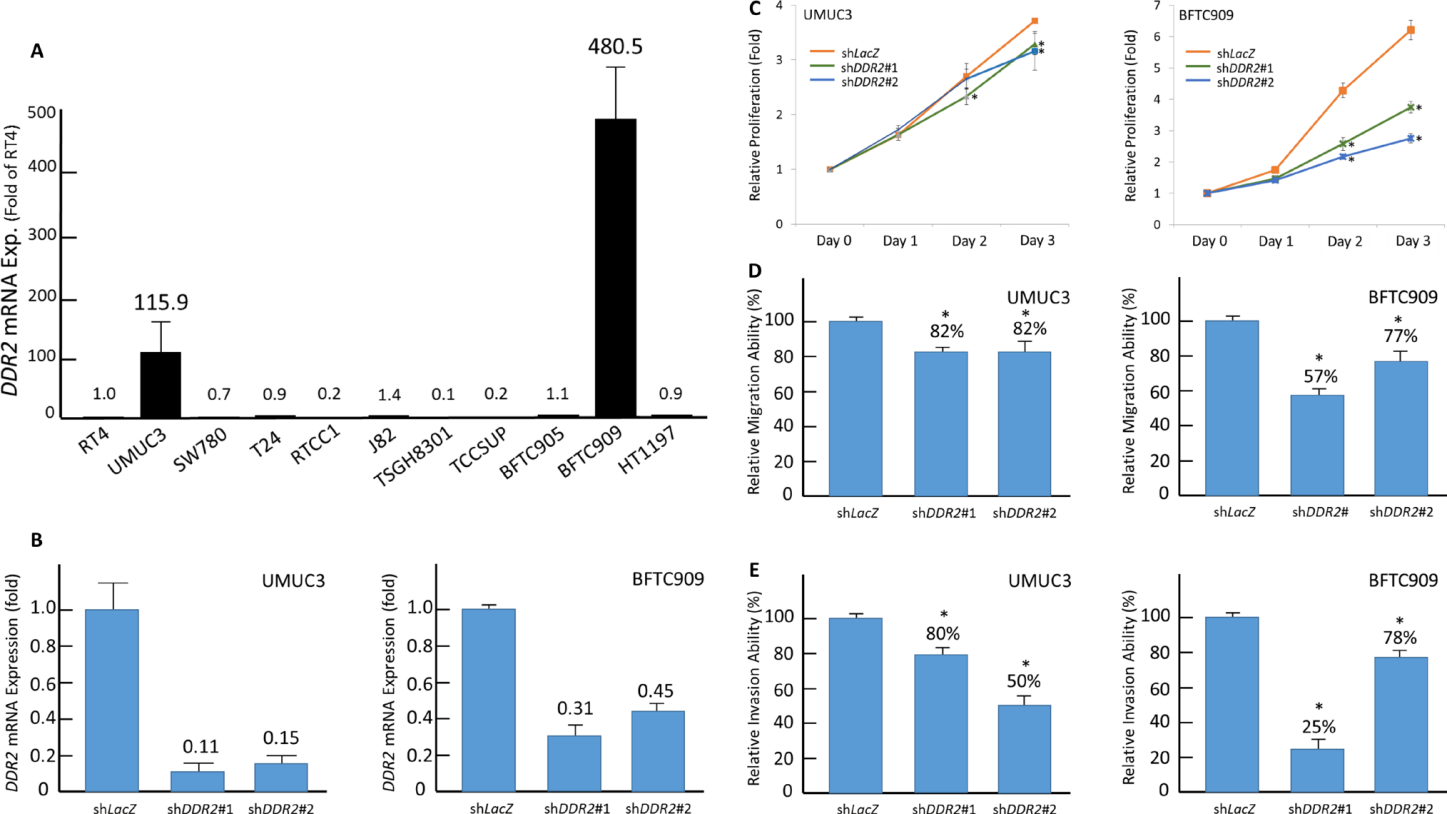
DDR2 expression is associated with tumorigenic potential by enhancing proliferative, migratory, and invasive ability of tumor cells (**A**) Endogenous *DDR2* transcript expression are determined by quantitative RT-PCR. Compared with grade 1 UC cell line RT4, there are two grade 3 UC cell lines showing high *DDR2* mRNA expression, including UMUC3 and BFTC909. (**B**) Both the high DDR2-expressing cell lines were subsequently infected by Lentivirus caring shRNA targeting DDR2 (*shDDR2*#1 and *shDDR2*#2). Successful knockdown of DDR2 was confirmed by quantitative RT-PCR. (**C**) Using 2,3-bis-(2-methoxy-4-nitro-5-sulfophenyl)-2H-tetrazolium-5-carboxanilide (XTT) assay to examined cell viability, we demonstrate positive effects of DDR2 expression on cell proliferation. Similar results are identified for cell migratory (**D**) and invasive ability (**E**). The quantified results are illustrated as means ± sd. Error bars show the standard error. Data represents mean values of three independent experiments. Student's *t-test* is used, (*P < 0.05).

DDR2 expression level was examined on urothelial cancer cell lines including RT4, SW780 (grade 1), TSGH8301 (grade 2), UMUC3, T24, J82, TCCSUP, BFTC905, BFTC909, and HT1197 (grade 3) [15, 52]. Real time PCR reveals high DDR2 mRNA expression in two grade 3 cell lines, UMUC3 and BFTC909 (Figure 6A). The expression ratios compared with the grade 1 cell line RT4 are 115.9 and 480.5, respectively. DDR2 knockdown was performed by using short-hairpin RNA (shRNA) which significantly decrease of *DDR2* mRNA expression in both cell lines infected by Lentivirus caring sh*DDR2*#1 and sh*DDR2*#2 (Figure 6B). As determined by using 2,3-Bis-(2-Methoxy-4-Nitro-5-Sulfophenyl)-2H-Tetrazolium-5-Carboxanilide (XTT) assay and modified Boyden chamber assay, both cell lines with depleted DDR2 expression revealed significant impaired proliferation, migration, and invasion ability, suggesting the role of DDR2 in promoting proliferative, migratory, and invasive ability of UC cell (Figure 6C and 6D).

## DISCUSSION

UC has four main altered molecular pathways, including the p53/Rb pathway, histone modification, RTK/Ras/PI(3)K pathway, and SWI/SNF complex, involving 93%, 89%, 72%, and 64% of the 131 UCs analyzed, respectively. The altered RTKs in UC have been reported and associated with tumor cell proliferation and survival, including FGFR3 activation, EGFR amplification, ERBB3 mutation, and ERBB2 mutation or amplification [10]. Due to the complex genetic alterations and multiple potential pathways of disease progression, the treatment for advanced UC has not progressed beyond cisplatin-based chemotherapy and surgery in the past 30 years, and there are no molecularly-targeted agents that have been approved [9, 10]. For the unmet need of therapeutic and prognostic biomarkers in UCs, several altered protein expression patterns in UC detectable by immunohistochemistry have been published, including IGFBP5, INHBA, CDCA5, FGF7, GPX2, NDN, SPOCK1, CEBPD, EMP2, *ZNF671, HSP90, and Gab1*. These proteins function in tumorigenesis, including the dysregulation of tumor growth, the avoidance of immune destruction, response to cellular stress, inhibition of apoptosis, and modification of the microenvironment of the extracellular matrix [4, 11–19]. DDR2 on the other hand, as a unique transmembrane receptor protein tyrosine kinase, promotes the epithelial mesenchymal transformation (EMT) and cellular migration through the blockade of extracellular matrix, thus enhancing the spreading of the malignancy. We demonstrated that DDR2 overexpression in UBUC and UTUC was significantly (Tables 3 and 4) and independently associated with an inferior prognostic outcome (Figure 2 and 5). The correlation is also proven by the mRNA transcription level (Figure 3). The reported “infiltrative” pattern rather than nodular or trabecular pattern, showing dismal prognosis in UC [51]. Our report revealed DDR2 overexpression significantly associating with infiltrative pattern (Table 2), supporting DDR2 stimulates EMT. The *in vitro* experiment performed in this study also supports this phenomenon (Figure 6).

DDR is a family of receptor tyrosine kinases originally isolated from normal human keratinocytes, resembling the Dictyostelium discoideum protein, discoidin [20]. Two groups of DDRs, DDR1 and DDR2, have been discovered and transcribed by chromosome 6 (6p21.3) and chromosome 1 (1q23.3), respectively [21, 22]. Unlike typical RTKs that bind soluble peptides, DDRs are activated by a variety types of human collagen in the triple-helical conformation, which is an extracellular matrix protein. The distinct preferences of DDR1 and DDR2 for various types of collagen have been investigated. DDR1 binds to collagen IV, V, VI and collagen VIII, while DDR2 binds to fibrillar collagen I, III, and X [22–24]. DDR1 and DDR2 bind to a specific amino acid motif (GVMGFO) in fibrillar collagen I-III and V. The different preferences arouse the hypothesis that cancer cells may hijack different DDRs to invade different extracellular circumstances and that they may serve as one of the cancer hallmarks: activating invasion and metastasis [25, 26]. DDR2 in cancer-associated fibroblasts was reported to influence tumor cell invasiveness by way of paracrine mechanisms, extracellular matrix production and remodeling, studied in breast cancer [27]. Upon binding to collagen, DDRs exhibit remarkably delayed (approximately 30 minutes) and sustained (up to 18 hours) receptor phosphorylation [22, 28]. The activated kinase domain of DDR2 then autophosphorylates several tyrosines in the proximal membranous region, which become docking sites for a variety of adaptor proteins, including SH2 domain-containing transforming protein 1 (Shc1), which is phosphorylated by DDR2 in a Src-dependent manner, resulting in the up-regulation of the promoter activity of matrix metalloproteinase-2 [29]. Two of the other adaptor proteins are extracellular signal-regulated kinase mitogen-activated protein kinase (ERK1/2-MAPK) and activator protein (AP)-1, related to DDR-2-mediated induction of the promoter activity of matrix metalloproteinase-13, which is involved in articular cartilage destruction in rheumatoid arthritis [30].

DDR2 has been studied in a variety of malignancies. DDR2 was mutated in 3–4% of squamous cell cancers of the lung [6], amplified in 10.4% and mutated in 2.2% adenocarcinomas of lung [31], amplified in 29% of neuroendocrine prostatic cancers [32], advanced prostatic adenocarcinomas [33], advanced hepatocellular carcinomas [34], nasopharyngeal cancers [35], amplified in 12.6% and mutated in 0.7% of invasive carcinomas of breast [36], and amplified in 10.1% of pancreatic adenocarcinomas [37]. Pure mutated DDR2 without evidence of an altered copy number was reported in a few malignancies including 13.8% of cutaneous squamous cell carcinomas [38], 6.9% of small cell lung cancers [39], 4.5% of uterine carcinosarcomas [40], and 4.1% of cutaneous melanomas [41]. Upregulation and mutations (R105S, H136H and N456S) of DDR2 in non-small cell lung cancer [42, 43] and mutations (G531V, S131C, T681I) of DDR2 in squamous cell carcinoma (SCC) of the lung have been reported. The mutation of S131C induces matrix metalloproteinases 2 and reduces E-cadherin expression of the lung SCC cells, promoting migration, invasion, plasticity, and EMT [44]. In hepatocellular carcinoma, invasiveness is assisted by DDR2 through activating ERK2 and stabilizing SNAIL1 [34]. In an invasive carcinoma of breast cancer, high DDR2 expression is significantly associated with a high tumor grade and triple-negative subtype and worse survival [42, 45]. In a xenograft model of gastric cancer, DDR2 overexpression is mediated by demethylation of a DNA promotor and knockdown of DDR2 suppressed peritoneal metastasis [46]. In UCs, the TCGA-documented UBUC displayed in cBioPortal for Cancer Genomic (http://www.cbioportal.org/, last updated in 2016/5/27) reveals that 14.5% of the 413 UBUCs have DDR2 gene amplification, and only 1.5% have DDR2 gene mutations [32]. In our study, DDR2 overexpression was assessed by the semiquantitive method of immunohistochemistry, showing increased protein expression associated with an advanced T stage and metastatic status, consistent with mainly copy number alterations found in UCs.

The concept that dysregulated the expression and/or function of RTKs involves cancer development, has been widely accepted. Several validated therapeutic targets to the RTKs have been developed, including trastuzumab to human epidermal growth factor receptor, cetuximab, erlotinib and gefitinib to epidermal growth factor receptor, etc. [47, 48]. Several studies on monoclonal antibodies, small chemical molecules interfering with intracytoplasmic activities, or blocking the interaction with collagen type I [25] further support that DDRs fasten cancer progression. Dasatinib, a multi-targeted TKI used to treat chronic myeloma, was proven to be an effective treatment for DDR2-mutated squamous cell carcinoma of the lung in xenograft models [43]. However, the clinical application was limited by toxicity, and a more selective DDR2 inhibitor compounded with an SRC inhibitor was developed and demonstrated enhanced suppression of DDR2-mutated lung cancer cell lines [44]. Actinomycin D, an anticancer agent, was also proven to disrupt the interaction between DDR2 and collagen in an *in vitro* study, without interfering with the activity of other receptor tyrosine kinases [9]. Some clinical trials are now recruiting study subjects. The trial for Regorafenib (NCT02795156), an inhibitor for a spectrum of RTKs, is currently recruiting patients with advanced cancer including UC and others with genomic alterations of VEGF, PDGFR, DDR2, etc. The trial for Nilotinib (NCT02029001) is recruiting patients with malignant solid neoplasms with mutations, amplification, or translocation of ABL1, KIT, PDGFRA, DDR1, DDR2, etc.

In conclusion, DDR2 overexpression is independently associated with tumor progression and dismal survival in UC patients. UC tumor cells may take advantage of DDR2 to enhance proliferation via EMT. Our study proposes that DDR2 is an important predictive marker for UC patients who are more vulnerable to disease progression and who may be a potential candidate for targeted therapy.

## MATERIALS AND METHODS

### Recognizing differentially expressed transcripts by data mining on gene expression omnibus

By performing data mining on the Gene Expression Omnibus database (National Center Biotechnology Information), we identified data set GSE31684 (http://www.ncbi.nlm.nih.gov/geo/query/acc.cgi?acc¼GSE31684) and investigated 93 UBUC specimens using the Affymetrix GeneChip Human Genome U133 Plus 2.0 Array. We imported the raw files into and computed the expression levels identified by probe sets, without preselection or filtering, using Nexus Expression 3 software (BioDiscovery, EI Segundo, CA, USA). We carried out a supervised comparative analysis to inspect the differentially-expressed genes with statistical significance according to the primary tumor status and metastatic status. We focused on the functional profiles of transcriptions associated with transmembrane receptor tyrosine kinase activity (GO:0004714). Further survival analysis was performed in all cases by dichotomize cases into high- and low-expression clusters in an unsupervised manner to computerize the prognostic impact of selected gene.

### Real-time RT-PCR

We assessed the *DDR2* transcript levels in snap-frozen samples bearing a high percentage of tumor components (70% at least) consisting of 26 UBUCs and 26 UTUCs. To achieve this goal, we extracted and submitted total RNAs for reverse transcription. As in our previous work using pre-designed TaqMan assay reagents (Applied Biosystems), we measured the mRNA abundance of *DDR2* (Hs01025953_m1) with the ABI StepOnePlus™ System. The expression ratio of *DDR2* relative to paired non-tumor urothelium was computed by a comparative Ct method, after normalization to *POLR2A* (Hs01108291_m1) as the internal control. [49]

### Case selection

For this study, we obtained approval from the institutional review board (IRB10302015) of the Chi Mei Medical Center. Based on the archives of the Chi Mei Medical Center during the interval of 1996 to 2004, we gathered 635 consecutively treated patients diagnosed with UC, including 340 with UTUC and 295 with UBUC. Cases were limited to “not otherwise specified” UC, excluding other variants. All patients received surgical treatment with curative intent. In UBUC, cisplatin-based adjuvant chemotherapy was administered for pT3 or pT4 tumors or for those with nodal metastasis. On the other hand, only 29 of 106 patients with advanced tumor stage (pT3 or pT4) or nodal metastatic UTUC underwent cisplatin-based adjuvant chemotherapy. As in our previous work, the clinicopathologic data for analysis included gender, age, multifocality, primary tumor stage, lymph nodal status, histological grade, vascular invasion, perineurail invasion, mitotic figures, and invasion pattern [11, 51]. Two pathologists (PIL & CFL) re-assessed the histologic features of all cases.

### Immunohistochemical staining and scoring of DDR2

Tissue sections underwent deparaffinization, rehydration and antigen retrieval. Subsequently, the sections were incubated with a primary antibody targeting DDR2 (1:100, H-108) for an hour. The primary antibodies were subsequently detected on the basis of the ChemMate DAKO EnVision kit (DAKO) [49]. DDR2 immunoreactivity scoring was evaluated by the H-score, which was generated from the following equation: H-score = Σ*P*_i_ (*i* + 1), where *i* represents the intensity of staining (0–3+), and *Pi* stands for the percentage of stained tumor cells (0% to 100%) [50].

### Generation of stable DDR2 expression cell lines

We purchased UC cell lines RT4, TSGH8301, TCCSUP, BFTC905, BFTC909 from the Food Industry Research and Development Institute of Taiwan. BFTC909 was derived from the rare sarcomatoid variant of UC in renal pelvis [52]. UMUC3, SW780, T24, J82, and HT1197 were purchased from American Type Culture Collection (Manassas, VA 20108, USA). RTCC1 derived from UC of the renal pelvis, was acquired from Professor Lien-Chai Chiang at Kaohsiung Medical University [53]. Cell culture condition was operated as recommended and described previously [15].

### RNA interference

We applied the Lentiviral expression plasmids purchased from the National RNAi Core Facility located at the Genomic Research Center of the Institute of Molecular Biology, Academia Sinica, Taiwan. The Lentivirus was cultured as suggested, and the viral supernatants were harvested in the conditioned medium. After confirming the efficiency of viral infection, we used these viral supernatants to infect the selected cell lines for 48 hours [49]. The shRNA sequences used in the vectors were: pLKO.1-*shLacZ* (TRCN000 0072223: 5′-TGTTCGCATTATCCGAACCAT-3′), pLKO.1-sh*DDR2*#1 (TRCN0000001418: 5′-GCC AGATTTGTCCGGTTCATT-3′; TRCN0000001419: 5′-GCCAAGTGATTCTAGCATGTT-3′).

### XTT (2,3-Bis-(2-Methoxy-4-Nitro-5-Sulfophenyl)-2H-Tetrazolium-5-Carboxanilide) assay

Cell viability was measured by XTT (Sigma) based on the product manual. Cells were set in 96-well plates at their appropriate concentrations (3,000~5,000cells/well). The cells were then incubated at 37°C in a humidified atmosphere containing 5% CO2. The culture medium was removed after 24, 48, 72 hours of incubation. An XTT reaction mixture was administered to each well and incubated for 4 four hours at 37°C. By the microplate reader, the absorbance was scored at a wavelength of 450 nm compared with a reference wavelength of 630 nm.

### Migration and invasion assays

Migration and invasion were studied via Boyden chamber technique (transwell analysis). The cell migration assay was performed using Falcon HTS FluoroBlok 24-well inserts (BD Biosciences). The cell invasion assay was done using the 24-well Collagen-Based Cell Invasion Assay (Millipore) [15].

### Statistical analysis

All analyses were carried out using SPSS V.14.0 software (SPSS Inc. Chicago, Illinois, USA). We dichotomized the study cohorts into high and low expression groups by using the median H-score of DDR2 immunoreactivity as the cut-off point. The high expression cohort was compared with the low expression cohort by performing a Chi-square test for clinicopathologic categorical variables. The survival statistics of interest were disease-specific survival (DSS) and metastasis-free survival (MeFS) up to 175 months of follow-up. Survival curves were sketched by the Kaplan–Meier method, and a log-rank test was performed to assess prognostic differences. Parameters with univariate *p-*values less than 0.05 were enrolled in multivariate tests conducted by Cox proportional hazards model. For all analyses, statistical significance was achieved by two-sided tests of significance with *p* < 0.05.

## Abbreviations

DDR2: Discoidin domain receptor 2
UC: Urothelial carcinoma
UBUC: urinary bladder urothelial carcinoma
UTUC: urinary tract urothelial carcinoma
RTK: receptor tyrosine kinase
DSS: disease-specific survival
MeFS: metastasis-free survival
RT-PCR: Reverse transcription polymerase chain reaction
EMT: epithelial-mesenchymal transformation
